# Pharmacodynamic evidence for tedizolid use in *Mycobacterium avium* lung disease

**DOI:** 10.5588/ijtldopen.25.0487

**Published:** 2026-01-09

**Authors:** D. Deshpande, S. Srivastava, T. Gumbo

**Affiliations:** 1 Baylor University Medical Center, Dallas, TX, USA;; 2 Division of Infectious Diseases, Department of Medicine, The University of Texas at Tyler School of Medicine, Tyler, TX, USA;; 3 Department of Cellular and Molecular Biology, University of Texas Health Science Center at Tyler, Tyler, TX, USA;; 4 Mathematical Modeling and AI Department, Praedicare Inc., Dallas, TX, USA;; 5 Hollow Fiber System and Experimental Therapeutics Laboratories, Praedicare Inc., Dallas, TX, USA;; 6 IMPI Biotechnology Company Incorporated, Harare, Zimbabwe.

**Keywords:** hollow fibre system, non-TB mycobacteria, pharmacokinetics, pharmacodynamics, Monte Carlo experiments

## Abstract

**OBJECTIVE:**

On guideline-based therapy for *Mycobacterium avium* complex (MAC) lung disease (LD), ∼50%–60% of patients suffer recalcitrant disease. Tedizolid is a promising agent based on the intracellular hollow fibre model of MAC-LD (HFS-MAC) using the MAC laboratory reference isolate (ATCC#700898), with a target exposure 0–24 area under the concentration-time curve (AUC_0–24_)-to-minimum inhibitory concentration (MIC) of 23.46.

**DESIGN:**

We performed a 28-day study in the HFS-MAC inoculated with five different MAC isolates treated with six daily exposures, using intrapulmonary pharmacokinetics of tedizolid. Data were analysed using inhibitory sigmoid maximal effect (*E*_max_) modelling of bacterial burden versus AUC_0–24_/MIC. Monte Carlo experiments were implemented to determine the optimal oral tedizolid dose achieving target exposure in lungs of 10,000 virtual patients.

**RESULTS:**

Tedizolid killed the five clinical isolates of MAC with an *E*_max_ of 3.61 ± 1.05 log_10_ CFU/mL below day 0 burden at a target exposure AUC_0–24_/MIC of 155.5 ± 38.84 (*r*^2^ = 0.98). In Monte Carlo experiments, the dose of 200 and 300 mg/day achieved the target exposure in 86% and 93% of patients, respectively.

**CONCLUSION:**

Based on the reported HFS-MAC studies using multiple clinical isolates of MAC, we propose that tedizolid at 200–300 mg/day should be tested as the backbone of novel combinations to treat MAC-LD.

*Mycobacterium avium* complex (MAC) lung disease (LD) constitutes 80% of all non-TB mycobacteria (NTM) lung disease, making it the main target to cure if morbidity and mortality from NTM is to be reduced.^1^ The pathology of MAC-LD is characterised by intracellular bacilli in the monocytic-lineage inside granulomatous lesions, at initial bacterial burdens (*B*_*0*_) of 4.2–6.2 log_10_ CFU/mL in cavitary disease.^2,3^ Clinical disease presents as bronchonodular or cavitary disease, with an average cavity diameter of ∼2 cm.^4^ American Thoracic Society/Infectious Diseases Society of America (ATS/IDSA) guidelines–based combination therapy (GBT) consists of a macrolide (clarithromycin or azithromycin), ethambutol, and a rifamycin (rifabutin or rifampin). However, only 43%–53% of patients achieve sustained sputum culture conversion (SSCC) at the end of 6 months of therapy; thus, majority of patients suffer recalcitrant disease.^5,6^ A mixture of pathological and clinical features predict response to therapy: i) *B*_*0*_ (akin to inoculum effect), ii) drugs with high intracellular penetration such as macrolides and macrolide-based regimens, and iii) presence of cavitary disease, especially iv) cavities of >2 cm diameter (due to reduced drug penetration).^4–10^ These factors, which represent general pharmacokinetic (PK) and pharmacodynamic (PD) parameters important in bacterial diseases, should be incorporated when developing PK/PD disease models for testing new drugs that could achieve higher SSCC than GBT.^10,11^

We developed a hollow fibre system (HFS) model of MAC-LD (HFS-MAC), which mimics i) intrapulmonary PKs of drugs and ii) the intracellular nature of MAC-LD and iii) bacterial burden, to study PK/PD of drugs, starting with inoculation with the standard MAC standard reference strain from the American Type Culture Collection (ATCC#700898).^11–16^ Subsequently, we selected five clinical isolates to inoculate the HFS-MAC.^14^ The five clinical MAC strains (2 *M. avium*, 3 *M. intracellulare*) were chosen for regulatory submission work because GBT in the HFS-MAC kill below *B*_*0*_ in 40% of the isolates reflects the 43%–53% SSCC encountered in clinical trials meta-analyses, and the bacterial kill slopes colony forming units (CFUs) per mL of GBT in the HFS-MAC with these isolates reflect those in patients sputa’.^14^ We have used the HFS-MAC to examine quinolones, tetracyclines, *β*-lactam antibiotics, and oxazolidinones to test if they could replace GBT drugs.^13–19^ In the HFS-MAC inoculated with ATCC#700898, we found that tedizolid has promising activity against MAC.^20^ However, regulatory agencies guidelines state that ‘∼4–5 organisms of the major target species or organism groups should be tested’.^21^ Therefore, in the present study, we investigated the use of tedizolid in the HFS-MAC inoculated with the five different clinical isolates.

Tedizolid exhibits several important PK parameters relevant to MAC-LD treatment. First, it achieves 10- to 15-fold concentration penetration into monocytes, demonstrating better potency for intracellular than extracellular pathogens.^22^ Second, after a dose of 200 mg of the prodrug, the active moiety achieves a plasma half-life of 9.23 h, a volume of distribution of 108.25 L, and a 0–24 h area under the concentration-time curves (AUC_0–24_) of 25.13 mg*h/L, with an epithelial lining fluid (ELF) half-life similar to plasma but an ELF-to-plasma AUC_0–24_ ratio of 40.^23^ This means that an AUC_0–24_ of 1,005.2 mg*h/L is achieved in ELF after 200 mg/day oral dose administration. Finally, tedizolid is available as both oral and intravenous formulations, making it ideal for treatment over many months of MAC-LD therapy.

## METHODS

ATCC#700898 was purchased from the ATCC (Manassas, VA, USA). Tedizolid phosphate powder (active moiety) was purchased from BOC Sciences (NY, USA). Hollow fibre cartridges were purchased from FiberCell (Frederick, MD, USA). Roswell Park Memorial Institute (RPMI) 1640 medium and heat-inactivated foetal bovine serum (FBS) were purchased from Sigma (St. Louis, MO). THP-1 monocytes were purchased from the ATCC (TIB-202). Bacterial stocks were prepared as published previously.^11–18,20,24^ For all experiments, isolates were thawed and then cultured in Middlebrook 7H9 broth supplemented with 10% oleic acid, albumin, dextrose, and catalase (OADC) for 4 days at 37°C to achieve logarithmic phase growth and then used in minimum inhibitory concentration (MIC) and HFS-MAC experiments.

### HFS-MAC studies

For MICs of the five isolates, we utilised the standard broth microdilution method using cation-adjusted Mueller–Hinton broth supplemented with 5% OADC.^25^ The construction details of the HFS-MAC have been published in numerous prior publications.^11–18,20,24^ Briefly, MAC-infected THP-1 cells were inoculated into the peripheral compartment of HFS-MAC units, with RPMI-1640 plus 2% heat-inactivated FBS as circulating fresh medium; according to the package insert, albumin in FBS is at most 50,000 mg/L. The protein concentration from 2% FBS in the HFS-MAC is low, with a final albumin concentration of 1,000 mg/L, close to human ELF concentrations. We mimicked the intrapulmonary PKs reported by Housman et al.^23^ in each HFS-MAC unit, at an ELF half-life of 9 h, to achieve ELF AUC_0–24_ of 0, 5, 10, 18, 20, and 50 mg*h/L, administered once daily for 28 days.^26^ Each isolate was treated with one tedizolid AUC/MIC exposure and had its own non-treated control. The central compartment of each HFS-MAC unit was sampled on day 28, just before dosing (0 h), and then at 1, 6, 7, 10, 12, 13, and 23.5 h after the dose, and concentrations were measured using methods published previously.^20^ The peripheral compartment was sampled for THP-1 cell and MAC CFU on days 0 (*B*_*0*_), 3, 7, 10, 14, 21, and 28. Viable THP-1 cell counts were determined using an automated cell counter (Sceptor, Millipore Sigma). Next, THP-1 cells were washed twice and lysed using phosphate-buffered saline plus 0.025% Tween-20 (PBS-T20), and the samples were serially diluted. The cultures were then spread on Middlebrook 7H10 agar and incubated for 14 days at 37°C under 5% CO_2_ for CFU counts.

### PK/PD modelling

Tedizolid HFS-MAC concentrations were modelled using a one-compartment model in ADAPT 5. We modelled the entire 28 days of dosing and PKs. The PK/PD target exposure was defined as the EC_80_ (exposure mediating 80% of *E*_max_).

### Monte Carlo experiments

The PK parameter estimates and variances published by Housman et al.^23^ were entered into subroutine PRIOR of ADAPT 5 software and are shown in Supplementary Data Table S1.^26^ While tedizolid protein binding is 86.1%–91.9%, the mean protein ELF/plasma ratio in adults is 0.13–0.25 for total protein and 0.1–0.19 for albumin (thus ∼3,500 mg/L), and mean tedizolid protein binding in ELF is expected to be negligible.^14,23,27,28^ Thus, we used the total concentrations measured in ELF.^23^ We generated serum and ELF PKs in 10,000 virtual patients treated with once daily oral tedizolid doses of 100, 200, 300, and 400 mg. We used the MIC range from our literature search, to identify the PTA at each MIC. The target was the average AUC_0–24_/MIC identified in our HFS-MAC with five isolates.

### PubMed search

Our aim was to find all literature related to the use of tedizolid in MAC-LD. We performed a PubMed search using the Medical Subject Headings (MeSH) of ‘*Mycobacterium avium*’ AND either ‘tedizolid’ or ‘TR-700’ or ‘DA-7157’. The last day of search was 8 February 2025. There was no exclusion of articles by language. We also examined the references cited by the papers for further publications not picked by PubMed.

## RESULTS

We searched the literature and identified six studies on tedizolid MIC distributions,^29–34^ one case study on use of tedizolid in MAC-LD, and two HFS-MAC studies with ATCC#700898.^20,24^ The MIC distribution results are shown in Table 1; the MIC_50_s differed by geographic location. The HFS-MAC using ATCC#700898 identified an EC_80_ AUC_0–24_/MIC of 23.46 and an *E*_max_ of 2.0 log_10_ CFU/mL.^20^ The single clinical case report is described in detail in the Discussion section.^35^

**Table 1.** Tedizolid MIC tests for *Mycobacterium avium* complex reported in the literature.

Year (reference)	*n*	Media	Method	Location	ATCC	0.25	0.5	1	2	4	8	16	32	64	MIC_50_	MIC_90_
2020^32^	51	CAMHB	BMD	Nijmegen, Netherlands	8	2	5	9	10	12	7	3	1	1	2	16
2017^33^	100	CAMHB	BMD	Tyler, Texas	1										8	>32
2022^29^	40	7H9	BMD	Shanghai, China	16	0	0	1	1	2	10	19	1	6	16	32
2022^30^	37	CAMHB	BMD	Cordoba, Spain		2	3	7	13	10	2				2	4
2022^31^	111	CAMHBT	BMD	Shanghai, China		0	7	6	6	20	43	18	4	7	8	16
2006^34^	13	CAMHB	BMD	Tyler, Texas											8	8
Total	352					3	15	23	30	44	62	40	6	14		

BMD = broth microdilution; CAMHB = cation-adjusted Mueller–Hinton broth; CAMHBT = CAMHB with TES buffer; 7H9 = Middlebrook 7H9 broth with 5% oleic acid–albumin–dextrose–catalase.

### HFS-MAC studies with five isolates

**Figure 1.**
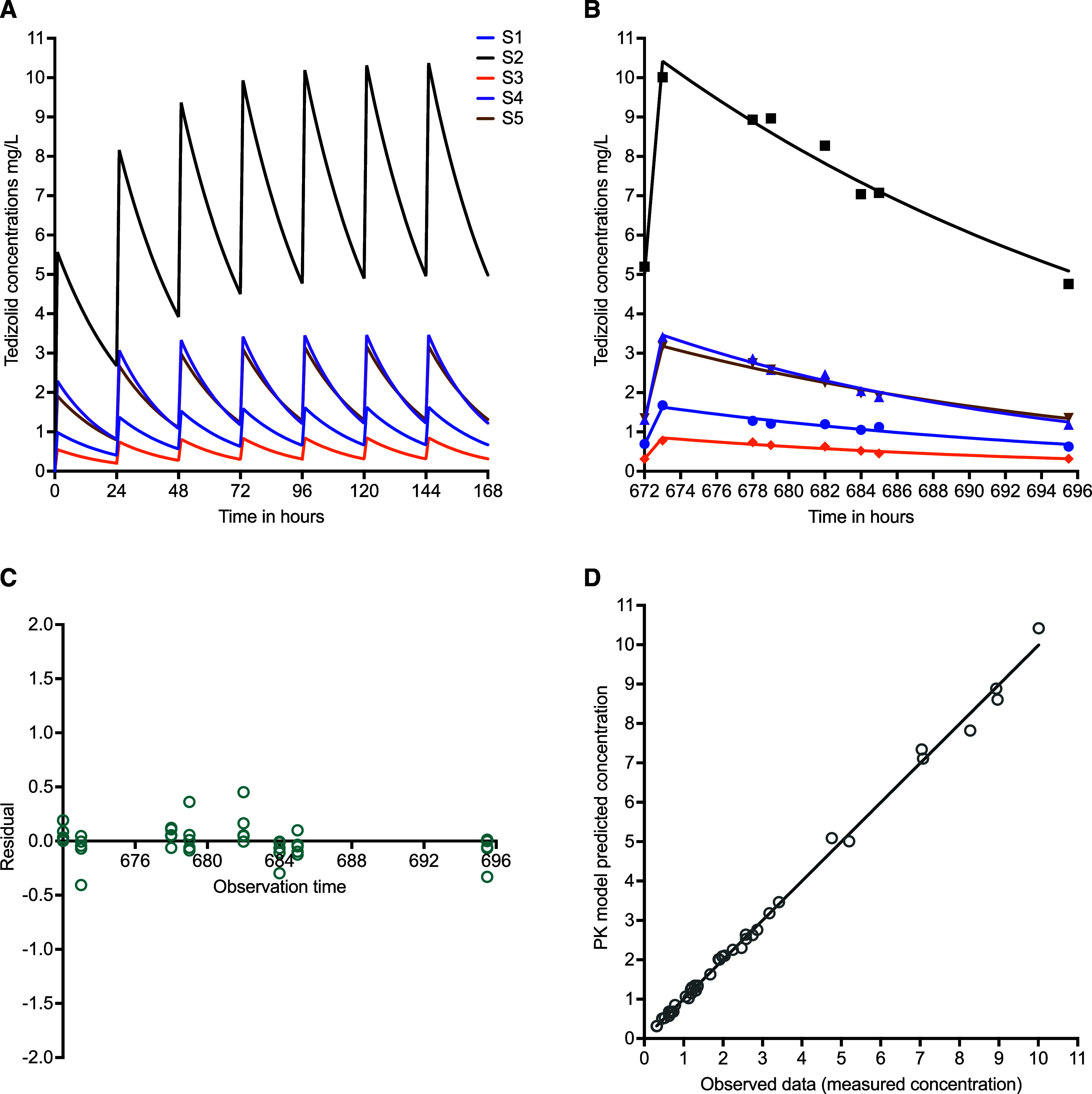
Measured tedizolid concentrations and pharmacokinetic modelling. **A:** Drugs measurements in each hollow fibre system unit were performed on day 28. Shown are the PK-modelled concentrations during the first 7 days to demonstrate steady accumulation in the HFS-MAC units. **B:** Shown is both model-predicted concentrations (line graph) and observed concentrations (symbols) on day 28 (672–696 h). **C:** Weighted residuals versus observation (sampling) time shows. **D:** PK model–predicted concentrations versus observed concentrations are depicted by symbols and line is a linear regression.

The tedizolid MICs for the five clinical isolates (S1–S5) were 0.125 mg/L for S1 and S2, 1 mg/L for S3 and ATCC#700898, 2 mg/L for S4, and 0.5 mg/L for S5. Figure 1 shows the tedizolid concentration-time profiles measured in the HFS-MAC units, equivalent to those in ELF. There was an accumulation of the drug in the HFS-MAC units, as demonstrated in the PK modelling output in Figure 1A and 1B. The PK model diagnostics are shown in Figure 1C and 1D. We identified a clearance of 0.016 ± 0.009 L*h^−1^ and a volume of 0.355 ± 0.263 L, with *r*^2^ for the HFS-MAC ranging from 0.944 to 0.987. The PK model–predicted versus observed concentrations are shown in Figure 1D, with a slope of 0.998 (95% confidence interval: 0.98–1.02) and *r*^2^ > 0.99, which indicates good model fit and no bias. The observed AUC_0–24_s were used to calculate AUC_0–24_/MIC.

**Figure 2.**
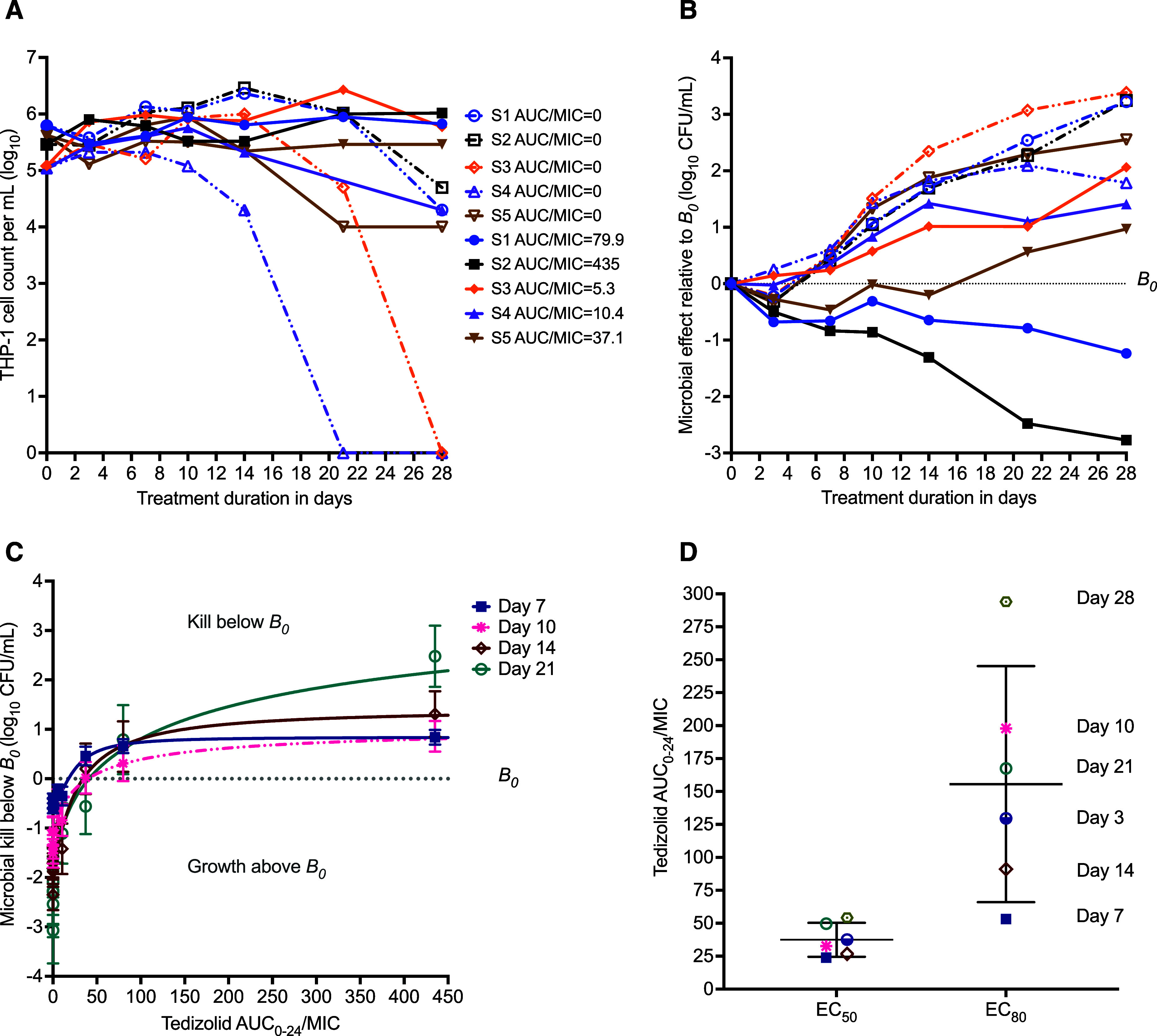
Tedizolid pharmacokinetics/pharmacodynamics in HFS-MAC with five clinical isolates. **A:** Shown are THP-1 cell mean counts in HFS-MAC units. Open symbols with hatched lines are non-treated systems (tedizolid AUC_0–24_/MIC = 0) and solid symbols with solid lines were treated with the AUC_0–24_/MIC exposures shown. Each symbol (triangle or square) has a corresponding non-treated system and a tedizolid-treated system. **B:** Changes in MAC burden on treatment with tedizolid normalised to B_0_. The symbols are for the regimens in panel A. **C:** Inhibitory sigmoid E_max_ curve for tedizolid exposures versus kill below *B*_*0*_ for different sampling days. For purpose of clarity and to avoid crowding, day 3 and 21 curves are not shown. **D:** The EC_50_s for each sampling day.

Even though the multiplicity of infection was the same for each of the five clinical isolates, the *B*_0_ achieved was different across the isolates, with the lowest as 60,000 CFU/mL and the highest as 540,000 CFU/mL. The effect of the different clinical isolates on the infected THP-1 monocytes in the HFS-MAC in treated and non-treated units is shown in Figure 2A. There were no MAC-free control HFS-MAC units. If we consider THP-1 cells killed by MAC as an indication of the virulence, the most virulent isolate was S4, which wiped out all THP-1 cells by day 21, while the least virulent isolate was S2, which only killed 0.77 log_10_ THP-1 cells/mL by day 28. Thus, we observed a heterogeneity in the virulence among the MAC clinical isolates.

Since the *B*_*0*_s were different for each isolate, the day-to-day changes are shown as kill below *B*_*0*_, in Figure 2B. Figure 2C shows inhibitory sigmoid *E*_max_ co-modelling of data from all five isolates versus tedizolid AUC_0–24_/MIC, on each sampling day. The model parameter estimates are shown in Table 2, which did not change outside the 95% confidence intervals between sampling days. The exposure mediating 50% of *E*_max_ (EC_50_) percent coefficient of variation (%CV) between sampling days was 32.69%, with the scatter shown in Figure 2D. The mean EC_80_ was an AUC_0–24_/MIC of 155.5 ± 34.84, which is the revised tedizolid target exposure to be achieved in the ELF of patients with MAC-LD. Sampling indicated a day-to-day increase in *E*_max_, and by day 28 it was 3.61 log_10_ CFU/mL below *B*_*0*_.

**Table 2.** Inhibitory sigmoid *E*_max_ parameter estimates and standard error (SE) from the HFS-MAC with five clinical isolates for pharmacodynamic endpoint of kill below *B*_*0*_.

	*E*_con_ log_10_ CFU/mL		*E*_max_ below B_0_ log_10_ CFU/mL		H		EC_50_ AUC_0–24_/MIC		*r* ^2^
	Estimate	SE	Estimate	SE	Estimate	SE	Estimate	SE	
Day 3	0.11	0.10	0.58	0.19	1.12	0.39	37.58	71.63	0.57
Day 7	−0.49	0.04	0.84	0.09	1.74	0.38	23.96	4.22	0.98
Day 10	−1.27	0.09	1.09	0.39	0.77	0.29	32.69	19.36	0.96
Day 14	−1.87	0.13	1.41	0.35	1.13	0.33	26.71	8.39	0.97
Day 21	−2.27	0.22	2.75	0.71	1.14	0.23	49.66	19.98	0.97
Day 28	−3.07	0.23	3.61	1.05	0.82	0.26	54.22	28.43	0.98

*E*_con_ = bacterial burden in non-treated controls reckoned log_10_ CFU/mL below *B*_*0*_; EC_50_ = exposure mediating 50% of maximal effect; *E*_max_ = maximal effect measured as log_10_ CFU/mL kill below *B*_*0*_; H = Hill slope.

### Monte Carlo experiments

**Figure 3.**
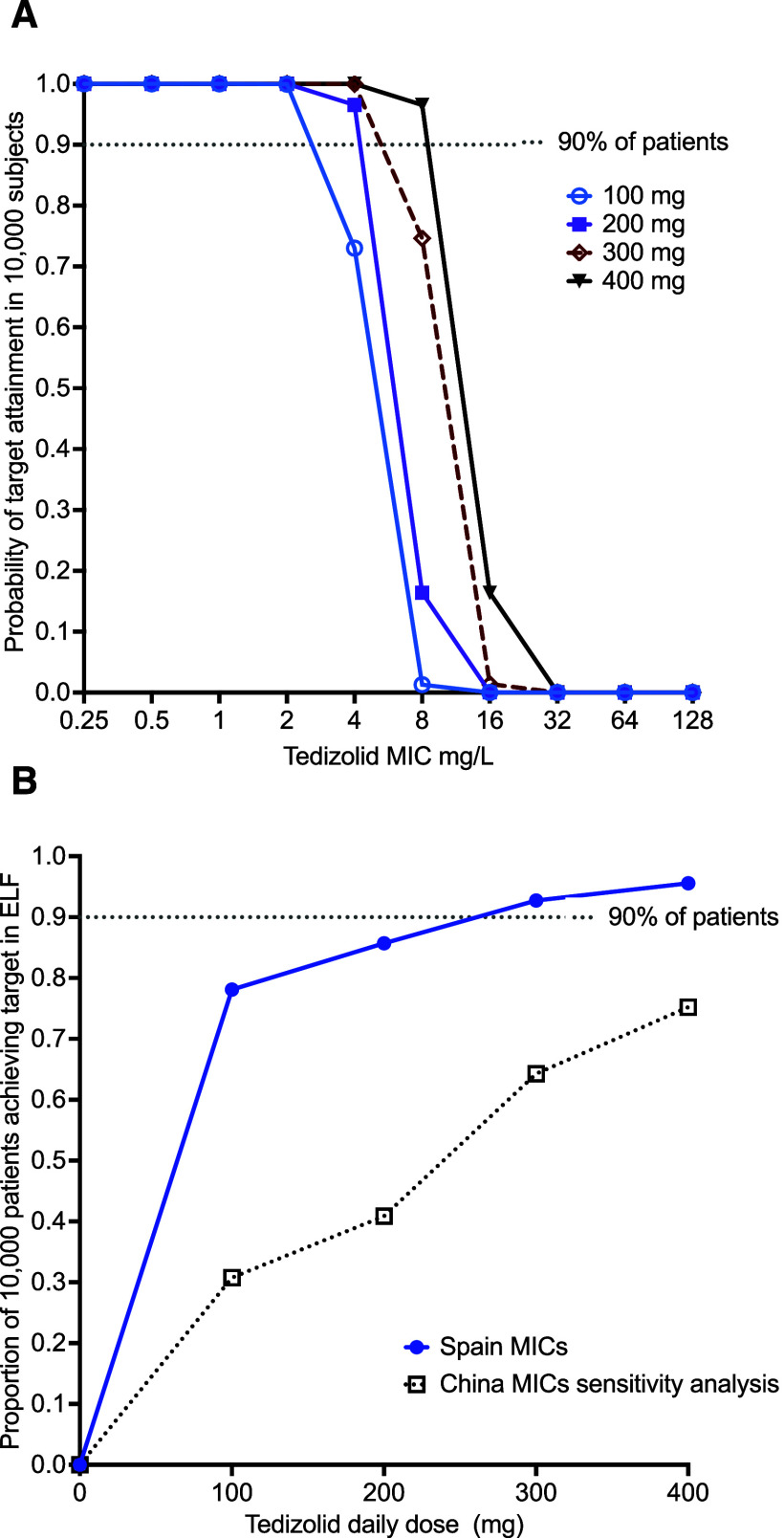
Monte Carlo experiments of 10,000 subjects treated with different tedizolid doses. **A:** Probability of target attainment (PTA) in 10,000 virtual subjects. **B:** The cumulative faction of response takes an expectation at each MIC and is thus sensitive to the MIC distribution.

The PK parameter estimates and variances in the 10,000 virtual subjects were compared to those in the domain of input in Supplementary Data Table S1, which shows that the Monte Carlo experiments recapitulated tedizolid population PKs well. As an example, the mean ± SD ELF AUC_0–24_ was 1,004 ± 252.9 mg*h/L for the 10,000 virtual subjects receiving the 200 mg/day dose versus 1,005 mg*h/L observed by Housman et al.^23^ The target exposure was the AUC_0–24_/MIC of 155.5. The probability of target attainment (PTA) at each MIC was as shown in Figure 3A. The PTA falls below 90% for the dose of 100 mg at an MIC of 4 mg/L, for 200 mg and 300 mg at an MIC of 8 mg/L, and for 400 mg at an MIC of 16 mg/L, suggesting a dose-dependent susceptibility breakpoint. At a dose of 200 mg/day, the PK/PD susceptibility breakpoint, which is the MIC at which the PTA falls below 90%, was 8 mg/L, while for a dose of 400 mg/day, it was identified as an MIC of 16 mg/L.

The cumulative fraction of response (CFR) is sensitive to the MIC distribution and is shown in Figure 3B for the MICs from Spain (MIC_90_ of 2 mg/L).^30,32^ The dose of 200 mg/day had a CFR of 86%, while that of 300 mg/day was 93%. On the other hand, a sensitivity analysis with the highest MIC distribution from China (MIC_90_ of 32 mg/L) demonstrated that the 400 mg/day dose would only achieve a CFR of 76%.^31^

## DISCUSSION

First, in the HFS-MAC using the ATCC#700898 reference strain from 8 years ago, we identified an EC_80_ AUC_0–24_/MIC of 23.46 versus an AUC_0–24_/MIC of 155.5 identified here.^20^ This is a multi-fold difference in potency and demonstrates that if ATCC#700898 alone is used in PK/PD studies, it could lead to misestimates of PK/PD target exposures. Indeed, microbial kill below *B*_*0*_ and PK/PD targets identified using ATCC#700898 alone have also led to misestimates when compared to parameters identified in the HFS-MAC inoculated with the five clinical isolates for i) GBT combination of macrolides, rifamycins, and ethambutol, ii) tetracyclines, and iii) ceftriaxone.^14,20^ Tedizolid efficacy (*E*_max_) with ATCC#700898 was 2.0 log_10_ CFU/mL versus 3.61 log_10_ CFU/mL below *B*_*0*_ identified here.^20^ Thus, use of multiple isolate likely leads to more accurate estimates of both PK/PD target exposure and efficacy.

Second, the most important use of PK/PD target exposures is the identification of the optimal dose that could be used in the clinic. Here, we identified robust PK/PD target exposure from five clinical isolates for Monte Carlo experiments. The PTAs together with the MIC distribution are crucial to conduct in silico dose-ranging experiments. The literature from Spain on MIC distribution of isolates showed that 200 mg/day had a CFR of 86% and 300 mg/day had a CFR of 93%.^30,32^ At these doses, the susceptibility breakpoint was an MIC of 8 and 16 mg/L, respectively. While technically 300 mg/day was >90%, in our assessment, 200 mg/day CFR of 86% is sufficiently close to 90% for us to recommend the lower dose, especially considering the better-known safety profile of the 200 mg/day tedizolid dose.

Third, we found a single case report, an example of real-world evidence, describing the use of tedizolid 200 mg/day dose in patients.^35^ Yuste et al.^35^ treated a patient in Pamplona, Spain, who was diagnosed with MAC and *M. kansasii* lung disease based on ATS/IDSA criteria. The two bacteria had clarithromycin, amikacin, and cotrimoxazole MICs demonstrating susceptibility. Linezolid MICs were 0.008 mg/L for MAC and 0.25 mg/L for *M. kansasii*, and tedizolid MICs were 0.002 mg/L for MAC and 4 mg/L for *M. kansasii*. The patient was treated with 600 mg twice daily linezolid, 15 mg/kg/day ethambutol, and 250 mg once a day of azithromycin. Linezolid was discontinued after 38 days due to toxicity and substituted with tedizolid 200 mg/day after a 26-day washout. Tedizolid treatment continued for 58 days, and subsequent clinical and chest CT scan results of the patient showed improvement. The case report provides real-world evidence of support for the dose, including safety.^35^

## CONCLUSION

We found that tedizolid exhibited significant microbial kill in the HFS-MAC against multiple clinical isolates. We revised the tedizolid PK/PD target exposure with a more robust and precise estimate. Monte Carlo experiments suggested that 200 mg/day dose should be sufficient for MAC-LD in large proportion of patients. Finally, the case report documents a clinical benefit and safety of tedizolid 200 mg/day combined with azithromycin and ethambutol for MAC-LD treatment.

## Supplementary Material




