# Supplementary material for: Pharmacodynamic evidence for tedizolid use in Mycobacterium avium lung disease
Source: IJTLD Open. 2026 Jan 9;3(1):17–23. doi: 10.5588/ijtldopen.25.0487 (PMC12810753; doi:10.5588/ijtldopen.25.0487)
Supplement: Supplementary file 1 [file ijtldopen25-0487_supplementarydata1.pdf]

Pharmacodynamic evidence for tedizolid use in *Mycobacterium avium* lung disease.

Devyani Deshpande[1], Shashikant Srivastava[1,2,3], Tawanda Gumbo[1,4,5,6]

[1]Baylor University Medical Center, Dallas, Texas, USA.

[2]Division of Infectious Diseases, Department of Medicine, The University of Texas at Tyler School of Medicine, Tyler, TX, USA.

[3]Department of Cellular and Molecular Biology, University of Texas Health Science Center at Tyler, Tyler, Texas, USA.

[4]Mathematical Modeling and AI Department, Praedicare Inc., Dallas, Texas, USA.

[5]Hollow Fiber System & Experimental Therapeutics Laboratories, NASOS Biotech, Dallas, Texas, USA.

[6]IMPI Group, Mt Hampden, Zimbabwe.

***Corresponding author:**

Tawanda Gumbo, MD

IMPI Group

29 Quinnington Road, Harare, Zimbabwe

mahwazhe@impigroup.com

Table S1. Monte Carlo experiments output compared to domain of input for pharmacokinetic parameters and variance.

	Domain of input based on publications[23,26]		10,000 virtual subjects	
	**Estimate**	**% IIV**	**Estimate**	**%IIV**
Total clearance [L/h]	8.46	25.0	8.45	24.97
Central Volume [L]	51.65	26.0	51.52	25.9
Intercompartmental clearance [$L*h^{-1}$]	0.973	34.0	0.97	33.83
Absorption constant [h^{-1}]	4.86	88.0	4.90	88.83
Peripheral volume [L]	15.06	59.0	15.05	58.75

*IIV=Inter-individual variability
